# Supplementary material for: Boosting the Humidity Performances of Na0.5BixTiO3 by Tuning Bi Content
Source: Nanomaterials (Basel). 2022 Jul 21;12(14):2498. doi: 10.3390/nano12142498 (PMC9315792; doi:10.3390/nano12142498)
Supplement: Supplementary file 1 [file nanomaterials-12-02498-s001.zip › nanomaterials-1803628-supplementary.pdf]

*nanomaterials*

*Supplementary Materials*

# Boosting the Humidity Performances of $Na_{0.5}Bi_xTiO_3$ by Tuning Bi Content

**Xiaoqi Xuan, Li Li, Tiantian Li, Jinsong Wang, Yi Yu * and Chunchang Wang ***

Laboratory of Dielectric Functional Materials, School of Materials Science & Engineering, Anhui University, Hefei 230601, China; b20201065@stu.ahu.edu.cn (X.X.); b20101003@stu.ahu.edu.cn (L.L.); b51914048@stu.ahu.edu.cn (T.L.); b20301123@stu.ahu.edu.cn (J.W.)
* Correspondence: 13070@ahu.edu.cn (Y.Y.); ccwang@ahu.edu.cn (C.W.)

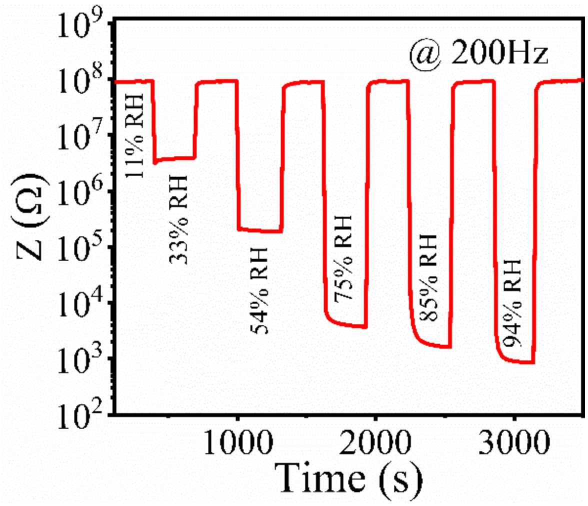


**Figure S1.** Dynamic response−recovery curve of the $Na_{0.5}Bi_{0.35}TiO_3$−based sensor between 11 % RH and other RHs.

**Table S1.** Response/recovery time of the $Na_{0.5}Bi_{0.35}TiO_3$−based sensor between 11% and other RH levels.

| RH level variation (% RH) | Response time (s) | RH level variation (% RH) | Recovery time (s) |
|---|---|---|---|
| 11→33 | 17.2 | 33→11 | 22.4 |
| 11→54 | 10.2 | 54→11 | 51 |
| 11→75 | 6.8 | 75→11 | 10.6 |
| 11→85 | 3.8 | 85→11 | 45.9 |
| 11→95 | 4.5 | 95→11 | 56.9 |

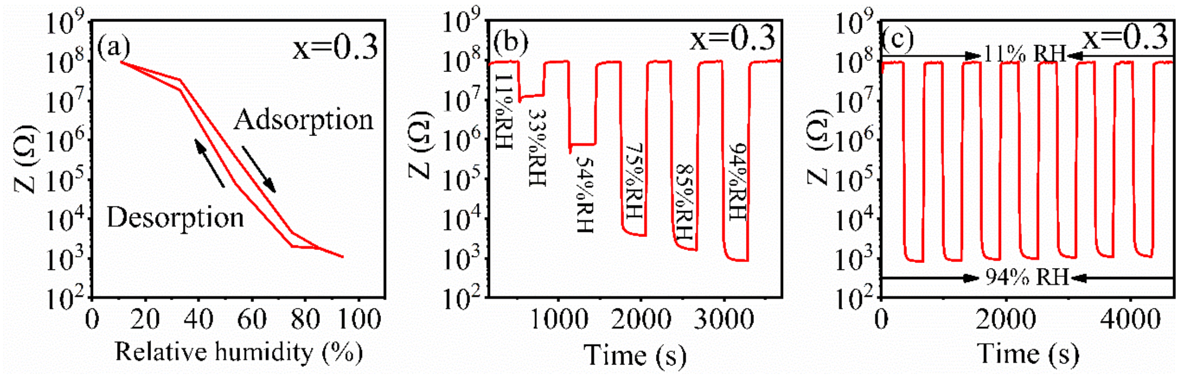


**Figure S2.** The humidity properties of $Na_{0.5}Bi_{0.3}TiO_3$–based sensor: (**a**) Hysteresis behavior, (**b**) response/recovery curve between 11 % RH and other RHs, and (**c**) repeatability curve.

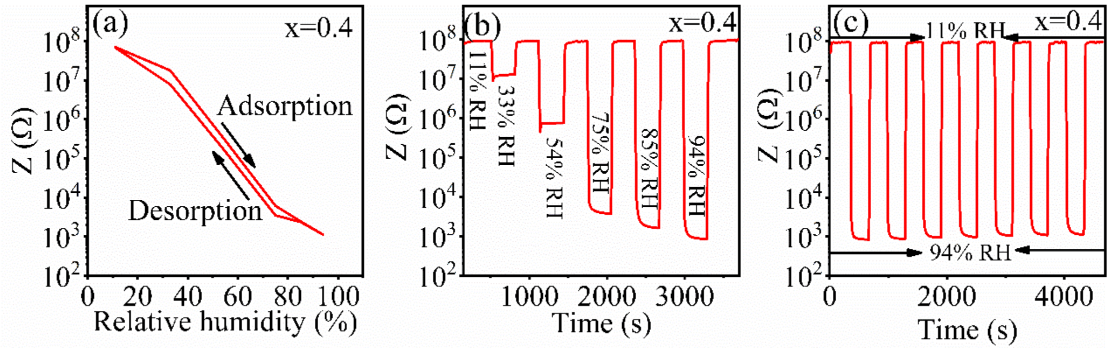


**Figure S3.** The humidity properties of $Na_{0.5}Bi_{0.4}TiO_3$–based sensor: (**a**) Hysteresis behavior, (**b**) response/recovery curve between 11 % RH and other RHs, and (**c**) repeatability curve.

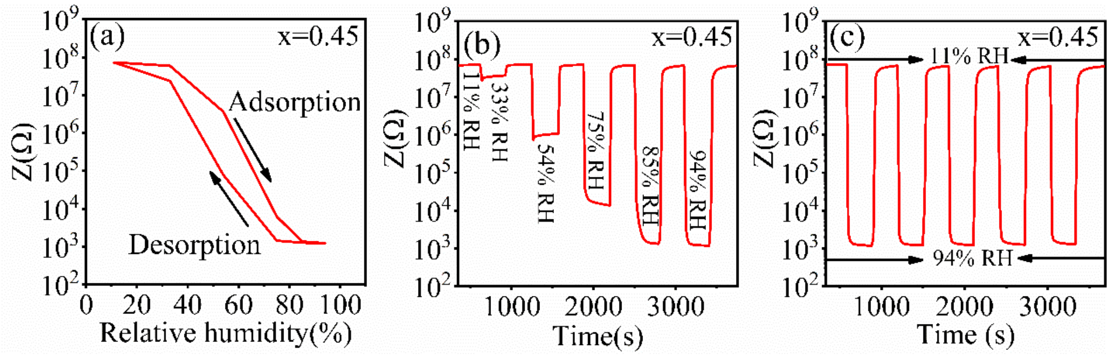


**Figure S4.** The humidity properties of $Na_{0.5}Bi_{0.45}TiO_3$–based sensor: (**a**) Hysteresis behavior, (**b**) response/recovery curve between 11 % RH and other RHs, and (**c**) repeatability curve.

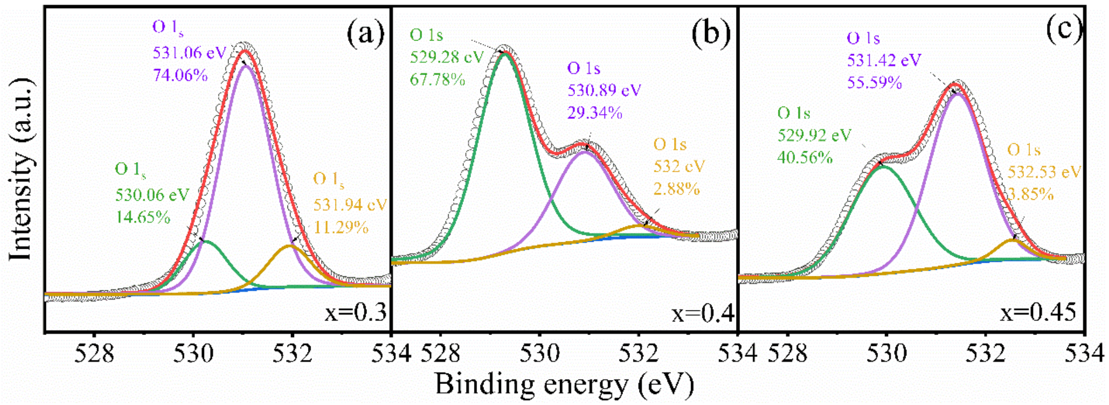


**Figure S5.** High−resolution XPS spectra of the O 1s (**a**–**c**) for the $Na_{0.5}Bi_xTiO_3$ (x = 0.3, 0.4, and 0.45).
